# Arginine improves the color stability of hemoglobin powder during freeze‐drying and storage

**DOI:** 10.1002/fsn3.1004

**Published:** 2019-03-31

**Authors:** Chengli Hou, Xuan Song, Zheng Li, Wenting Wang, Qingwu Shen, Dequan Zhang

**Affiliations:** ^1^ Institute of Food Science and Technology, Chinese Academy of Agricultural Sciences/Key Laboratory of Agro‐Products Processing Ministry of Agriculture and Rural Affairs Beijing China; ^2^ College of Food Science and Technology Hunan Agricultural University Changsha Hunan China

**Keywords:** arginine, blood, color, hemoglobin, stability

## Abstract

To increase the color stability of hemoglobin (Hb) powder, the technological conditions for arginine–hemoglobin (Arg‐Hb) powder preparation were optimized by response surface methodology and the influence of arginine (Arg) on the color stability of Hb powder was evaluated. Results showed that: (a) Arg‐Hb powder had better colors (less MetHb% and higher a* value) than Hb powder (*p* < 0.05); (b) using MetHb% as an indicator, the optimal conditions to prepare Arg stabilized Hb were Arg concentration of 10.5 mg/ml Hb extract, reaction pH of 10.75, and reaction temperature of 18°C; (c) pH and NaCl had a significant influence on the color stability of Hb (*p* < 0.05). At various NaCl concentrations and pH conditions, Arg‐Hb solution showed better color than Hb (*p* < 0.05); (d) Arg‐Hb powder had higher a* values and higher percentages of deoxyhemoglobin and oxyhemoglobin but lower MetHb% than Hb powder during storage (*p* < 0.05).

## 1 INTRODUCTION

Animal blood is an abundant coproduct after animals were slaughtered. The annual yield of animal blood is about 10 million tons in the world (Liu, Wu, Zhang, Wan, & Zhao, 2012). Currently, most processed animal blood is used in feed with a low commercial value. Technologies are needed for high‐value utilization of animal blood. For example, the production of colorant increases the values of animal blood and economic benefits of industry (Sakata, Lee, & Nagata, 1992). Blood is composed of plasma (~65%) and blood cell fractions (~35%). Hemoglobin (Hb) is an iron‐containing molecule present in red blood cells (Fontes, Gomide, Fontes, Ramos, & Ramos, 2010). Fresh Hb can be considered as a natural red colorant, but the redness is unstable during processing and storage as the ferrous iron in Hb is easy to be oxidized to ferric iron in vitro (Salvador, Toldrà, Parés, Carretero, & Saguer, 2009). The color of Hb depends on the redox state of the heme iron. Deoxyhemoglobin (DeoxyHb) exhibits a purple‐red color. Oxyhemoglobin (HbO_2_) has a bright red color. Methemoglobin (MetHb) with a ferric iron is in an undesirable brown color (Damodaran, Parkin, & Fennema, 2007).

For industrial application, Hb with greater color stability is needed. Many methods have been developed to increase the stability of Hb. For example, carbon oxide (CO) and nitric oxide (NO) have been used to prevent the oxidation and enhance the color stability of Hb, which combine to heme iron in Hb to replace oxygen (Fontes et al., 2010; Shahidi & Pegg, 1991; Zhang, Li, Kong, & Liu, 2013). However, both CO and NO are toxic, which challenge its use in Hb processing (Foresti, Bani‐Hani, & Motterlini, 2008). Some other agents, such as nicotinic acid, nicotinamide, cysteine, l‐histidine, and l‐arginine (Arg), have been reported to retard the oxidation and browning of Hb (Saguer et al., 2003; Salvador et al., 2009; Zhou, Wang, Chen, & Chen, 2012; Zhou, Ye, Nishiumi, Qin, & Chen, 2014; Zhou, Ye, Wang, Qin, & Li, 2015). Nicotinic acid and nicotinamide are able to form complexes with Hb. The formed complexes are stable during spray drying (Saguer et al., 2003; Salvador et al., 2009). In addition, cysteine, histidine, and arginine enhance the stability of Hb concentrates by coordinating with free iron (Zhou, Wang, Chen, et al., 2012; Zhou, Wang, Wang, Zhang, & Cai, 2012).

Recently, Zhou et al. (2015) reported that arginine improved the stability of liquid hemoglobin by binding free ion, but the influence of Arg on the color stability of Hb powder, a more common product of Hb, is unknown. In the present study, the technological conditions for the production of Arg stabilized Hb (Arg‐Hb) powder were optimized based on the value of MetHb%. The color stability of Arg‐Hb powder during storage was evaluated under different application conditions.

Currently, Hb powder is mainly utilized in meat products to enhance meat color and more of Hb powder treated with protecting agents to make Hb more useful as a natural colorant (Ofori & Hsieh, 2012). Arg itself is not toxic, and using as a supplement is generally safe for human (McKnight et al., 2010). The research results can provide data support for industrial processing of hemoglobin colorants.

## 2 MATERIALS AND METHODS

### 2.1 Materials

Sheep blood was collected from a local slaughterhouse. Hb standard was purchased from YuanYe Biotechnology Co., Ltd. (Shanghai, China). l‐Arginine was purchased from Aladdin Biochemical Technology Co., Ltd. (Shanghai, China). Other chemicals were purchased from Sinopharm Chemical Reagent Co., Ltd. (Beijing, China). All chemicals were of analytical grade.

### 2.2 Preparation of Hb

Hemoglobin was prepared according to the method described by Saguer et al. (2003) with some modification. Blood was collected, immediately mixed with sodium citrate solution (5.0% w/v) at a blood/solution ratio of 9:1 (v/v), and transported at 4–7°C to laboratory in plastic bottles. Red blood cells were collected by centrifugation at 8,000 *g*, 4°C for 20 min (Himac CR22 GⅡ; Hitachi, Ltd., Tokyo, Japan) and then washed three times with 0.9% (w/v) NaCl. Washed red blood cells were added with two volumes of ultrapure water, sonicated (KH7200DE; Hechuang Ultrasonic Instrument Co., Ltd., Jiangsu, China) for 15 min, and incubated at 60°C for 30 min in the dark. Hb in the supernatant (Hb extract) was collected after centrifugation and stored at 4°C until further processing.

### 2.3 Preparation of Arg‐Hb powder

The effects of Arg concentration, pH, temperature, and reaction time on producing Arg‐Hb were evaluated by the color measurement and the relative percentage of Hb derivatives.

Prepared Hb extract was added with 0, 2, 4, 6, 8, 10, or 12 mg/ml l‐Arg and incubated. The effects of Arg concentrations, reaction pH (9.5, 10, 10.5, 11, 11.5, and 12), temperature (5, 15, 25, 35, and 45°C), and time (5, 10, 15, 20, and 25 min) on the color stability of Arg‐Hb were evaluated by the color and relative content of Hb derivatives in Arg‐Hb powder. After the reaction, samples were lyophilized (LGJ‐25; Four‐Ring Science Instrument Plant Beijing Co., Ltd, Beijing, China) under the following conditions: cold trap temperature, −60°C; materiel temperature, −40°C, vacuum degree, 1 Pa. After single‐factor tests, a central composite design comprising three variables (Arg concentration, temperature, and pH) and 17 independent runs were conducted to optimize the technological conditions for the production of Arg‐Hb powder with a minimum MetHb%. The Arg concentrations varied from 8 to 12 mg/ml, the reaction temperature varied from 15 to 35°C, and the reaction pH ranged from 10 to 12 in the central composite design experiment.

### 2.4 Objective color measurement

The Hb powders were placed in transparent plastic plate (35 mm diameter). The surface color (CIE‐L*a*b*) of Hb powder was measured using digital color imaging system (DigiEye) (Verivide, Co., Ltd, Leicester, UK) (Jørgen, Leif, Marianne, & Birkeland, 2011). The DigiEye Cube on the DigiEye equipment was utilized to control light with diffuse illumination of illuminant D65. A digital camera Nikon D90 was centrally positioned on top of the cube and captured the high‐resolution images. The DigiEye software was used to given L*, a*, b* values according to the CIELAB definition.

### 2.5 Analysis of Hb derivatives

The percentage of Hb derivatives in Arg‐Hb powder was measured according to Benesch, Benesch, and Yung (1973) method with some modification. Arg‐Hb powder was dissolved in ultrapure water to a final concentration of 1 mg/ml. Samples were scanned from wavelength 650 to 500 nm using a UV‐1800 spectrophotometer (Shinadzu Co., Ltd., Suzhou, China). The HbO_2_, DeoxyHb, and MetHb contents were calculated based on the absorbance at 560, 576, and 630 nm according to the following equations:

$$\left[{\text{HbO}}_{2}\right]=\left(1.013\times {\text{Abs}}_{576}-0.3269\times {\text{Abs}}_{630}-0.7353{\text{Abs}}_{560}\right)\times 10^{-4}$$

$$\left[\text{DeoxyHb}\right]=\left(1.373{\text{Abs}}_{560}-0.747\times {\text{Abs}}_{576}-0.0737\times {\text{Abs}}_{630}\right)\times 10^{-4}$$

$$\left[\text{MetHb}\right]=\left(2.985\times {\text{Abs}}_{630}+0.194\times {\text{Abs}}_{576}-0.4023\times {\text{Abs}}_{560}\right)\times 10^{-4}$$

The relative content of Hb derivatives was expressed as a percentage of the total Hb.

### 2.6 The effect of pH and NaCl on the stability of Arg‐Hb

To examine the stability of produced Arg‐Hb powder, samples were dissolved in pH buffers or NaCl solutions. pH buffers (pH 5, 6, 7, 8, 9, 10, 11, and 12) were prepared by adjusting the pH of 0.5 M sodium dihydrogen phosphate with 2 M NaOH. NaCl solutions (0, 0.2, 0.4, 0.6, 0.8, and 1.0 mol/L) were prepared by directly dissolving NaCl in distilled water. Arg‐Hb powder was dissolved in sodium phosphate buffers or NaCl solutions to a final concentration of 1 mg/ml. The solutions were centrifuged (10,000 *g*, 10 min). The absorbance of supernatant at 540 nm was measured.

### 2.7 Stability of Arg‐Hb powder during storage

Response surface methodology showed that Arg‐Hb powder prepared under the conditions of 10.5 mg/ml Arg, reaction pH 10.75, temperature 28°C, and reaction time of 10 min had the lowest MetHb content. Arg‐Hb powder prepared under these conditions was further used for further analysis of the color stability of Arg‐Hb during storage. Arg‐Hb powder was stored at room temperature and analyzed on days 0, 1, 3, 5, and 7. Hb sample without addition of Arg was used as control.

### 2.8 Statistical analysis

The central composite design was set up using Design‐Expert 7.0.0 (Stat‐Ease, Inc., Minneapolis, MN, USA). Statistical analyses were performed using SPSS 17 (SPSS Inc., Chicago, IL, USA). Values were presented as mean ± *SD*. The differences between the Arg‐Hb and control groups were assessed by a paired *t* test. *p* values < 0.05 were considered significant. Unless stated otherwise, statistical comparisons were made using one‐way analysis of variance (ANOVA). The differences in means were evaluated by the Student–Newman–Keuls test (*p* < 0.05).

## 3 RESULT AND DISCUSSION

### 3.1 Impact of Arg concentration, reaction temperature, time, and pH on the stability of Hb

**Figure 1:**
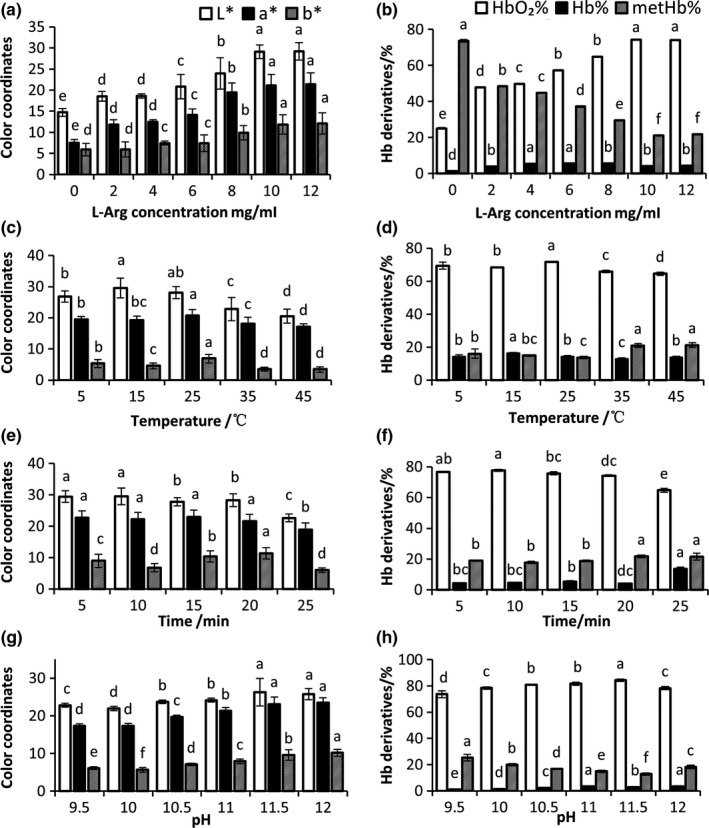
(a, b) Impact of l‐Arg concentration on color coordinates and Hb derivatives. (c, d) Impact of reaction temperature on color coordinates and Hb derivatives. (e, f) Impact of reaction time on color coordinates and Hb derivatives. (g, h) Impact of reaction pH on color coordinates and Hb derivatives. Different letters represent significant differences at *p < *0.05. Data are presented as mean ± *SD*,* n* = 3

To investigate the influence of Arg concentration, reaction temperature, time, and pH on the stability of Hb, the surface color (CIE‐L*a*b*) of and the forms of Hb in prepared Hb powder were determined. As shown in Figure 1a, Arg had a significant influence on the color of Hb powder (*p* < 0.05). The L* and a* values of Arg added samples were higher than those of control (*p* < 0.05). Accordingly, Arg significantly affected the transformation of different Hb forms (Figure 1b). Compared with the control, Arg‐treated samples had higher HbO_2_% and lower MetHb% (*p* < 0.05). The effect of Arg on the color of Hb powder was concentration dependent. The L* value, a* value, and HbO_2_% increased with the increase of Arg concentration in the range of 0 to 10 mg/ml. When Arg concentration was higher than 10 mg/ml, a* value and HbO_2_% did not increase anymore (*p* > 0.05). However, a* value and HbO_2_% in Arg treatment group were significantly higher than that in control (*p < *0.05), while MetHb% was higher in control than that in Arg treatment. In summary, the results showed that Arg prevented the oxidation of Hb and improved the color of Hb powder, which is consistent with the previous study (Zhou et al., 2015). This might be owing to the reducing property of l‐Arg (Nigris et al., 2003).

As shown in Figure 1c,d, reaction temperature significantly influenced the color of the Arg‐Hb powder as well as the forms of Hb. The L* values of samples treated at 15 and 25°C were higher than that of other treatments (*p < *0.05). The a* values were not different among 5, 15, and 25°C treated samples. When the reaction temperature further increased from 25 to 45°C, a* values of Hb powder decreased (*p < *0.05). In addition, HbO_2_% in Hb powder increased with the increase of reaction temperature, which maximized at 25°C. When reaction temperature was higher than 25°C, HbO_2_% in Hb powder decreased with the increase of reaction temperature. All these results show that the optimal reaction temperature for Arg‐Hb powder preparation is about 25°C.

As shown in Figure 1e,f, the samples treated for 5 or 10 min had higher a* value and HbO_2_% than other treatments. When reaction time increased from 10 to 25 min, MetHb% in Hb powder increased and HbO_2_% decreased. All these results show that 5‐ to 10‐min reaction time is enough for Arg to react with Hb in the preparation of Arg‐Hb powder.

As shown in Figure 1g,f, reaction pH significantly influenced the color of Arg‐Hb powders (*p* < 0.05). The sample treated at pH 11.5 has the highest a* value and HbO_2_%, and the lowest MetHb%. High pH denatures Hb (Rieder, 1970), making it easier for oxygen molecules to access heme iron and form HbO_2_ (Winslow, 2000). This is also observed by others (Zhou et al., 2015).

### 3.2 Optimal reaction conditions for Agr‐Hb powder preparation

**Figure 2:**
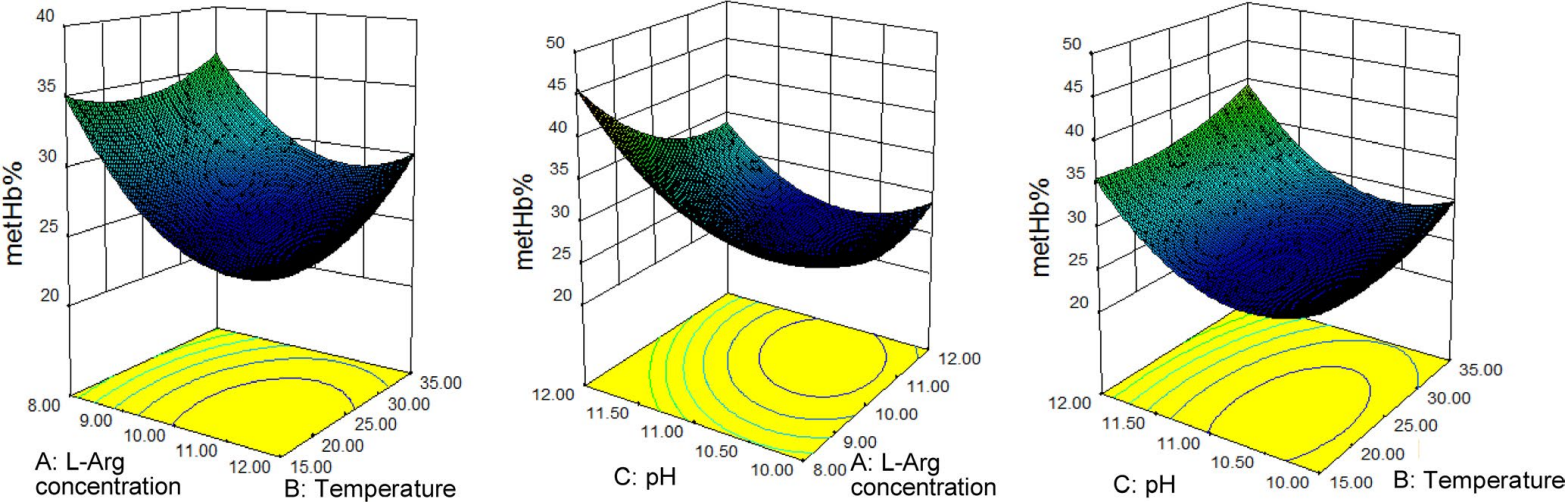
Response surface plots for interactive effects of l‐Arg concentration, temperature, and pH on MetHb%

The reaction conditions including Arg concentration, reaction pH, and temperature for Arg‐Hb powder preparation were optimized by response surface methodology. The combined effects of l‐Arg concentration (X_1_), reaction temperature (X_2_), and pH (X_3_) on MetHb% (Y) are presented in Table 1. Table 2 shows the coefficients of variables and statistical significances evaluated by ANOVA test. Statistical parameters such as coefficient (*R* ^2^) and *F* test probability are also summarized in Table 2. The result for the *R* ^2^ values is 0.9588. The model for Arg‐Hb is as follows: Y = 25.98 − 4.03X_1_ + 1.55X_2_ + 4.40X_3_ + 0.94X_1_X_2_ − 1.93X_1_X_3_ + 0.29X_2_X_3_ + 3.93X_1_^2^ + 1.67X_2_^2^ + 5.55X_3_^2^. The impact order of the factors is as follows: pH > Arg concentration > temperature. As shown in Figure 2, interactions among the reaction parameters, Arg concentration, pH, and temperature, were determined. Response surface plots showed that the order of the interaction is as follows: pH and Arg concentration > Arg concentration and temperature > temperature and pH. However, there were no significant interactions between any of the two factors by ANOVA. The value 0.0734 for interactive coefficient is an indication of interaction in Arg concentration and pH.

**Table 1:** Central composite design matrix and the experimental results of MetHb% under different preparation conditions

Run	Independent variables^a^						Dependent variable (MetHb%)
	Coded level			Uncoded level			
	X_1_	X_2_	X_3_	A	B	C	
1	0	0	0	10	25	11	25.98
2	−1	0	1	8	25	12	46.56
3	0	0	0	10	25	11	25.98
4	−1	0	−1	8	25	10	33.27
5	−1	−1	0	8	12	11	36.21
6	0	1	−1	10	35	10	32.00
7	1	0	1	12	25	12	33.78
8	0	0	0	10	25	11	25.98
9	0	0	0	10	25	11	25.98
10	1	0	−1	12	25	10	28.21
11	0	−1	1	10	12	12	33.81
12	0	0	0	10	25	11	25.98
13	1	1	0	12	35	11	28.84
14	−1	1	0	8	35	11	34.16
15	0	1	1	10	35	12	40.76
16	0	−1	−1	10	15	10	26.23
17	1	−1	0	12	15	11	27.12

a Independent variables: X_1_ and A, l‐Arg concentration (mg/ml); X_2_ and B, reaction temperature (^o^C); X_3_ and C, reaction pH.

**Table 2:** Analysis of variance of the quadratic regression model

Factor	Sum of squares	Degrees of freedom	Mean square	*F*‐value	*p*‐value	Significance
Model	548.11	9	60.90	18.10	0.0005	**
X_1_	130.01	1	130.01	38.64	0.0004	**
X_2_	19.19	1	19.19	5.70	0.0483	*
X_3_	154.88	1	154.88	46.03	0.0003	**
X_1_X_2‐_	3.55	1	3.55	1.06	0.3383	
X_1_X_3_	14.90	1	14.90	4.43	0.0734	
X_2_X_3_	0.35	1	0.35	0.10	0.7571	
X_1_^2^	64.99	1	64.99	19.32	0.0032	**
X_2_^2^	11.80	1	11.80	3.51	0.1033	
X_3_^2^	129.52	1	129.52	38.49	0.0004	**
Residual	23.55	7	3.36	–	–	
Lack of fit	23.55	3	7.85	–	–	
Pure error	0.00	4	0	–	–	
Cor total	571.67	16	–	–	–	

* *p* < 0.05

** *p* < 0.01

Optimum reaction conditions (Arg concentration, pH, and temperature) regarding lowest MetHb% were predicted to be Arg concentration 10.5 mg/ml, pH 10.75, and reaction temperature 18°C. Under these conditions, predicted value for MetHb% was 24.19%. The practical result for MetHb% was 23.36%±1.72%, which was close to the prediction.

### 3.3 Effect of NaCl and pH on the stability of Arg‐Hb powder

**Figure 3:**
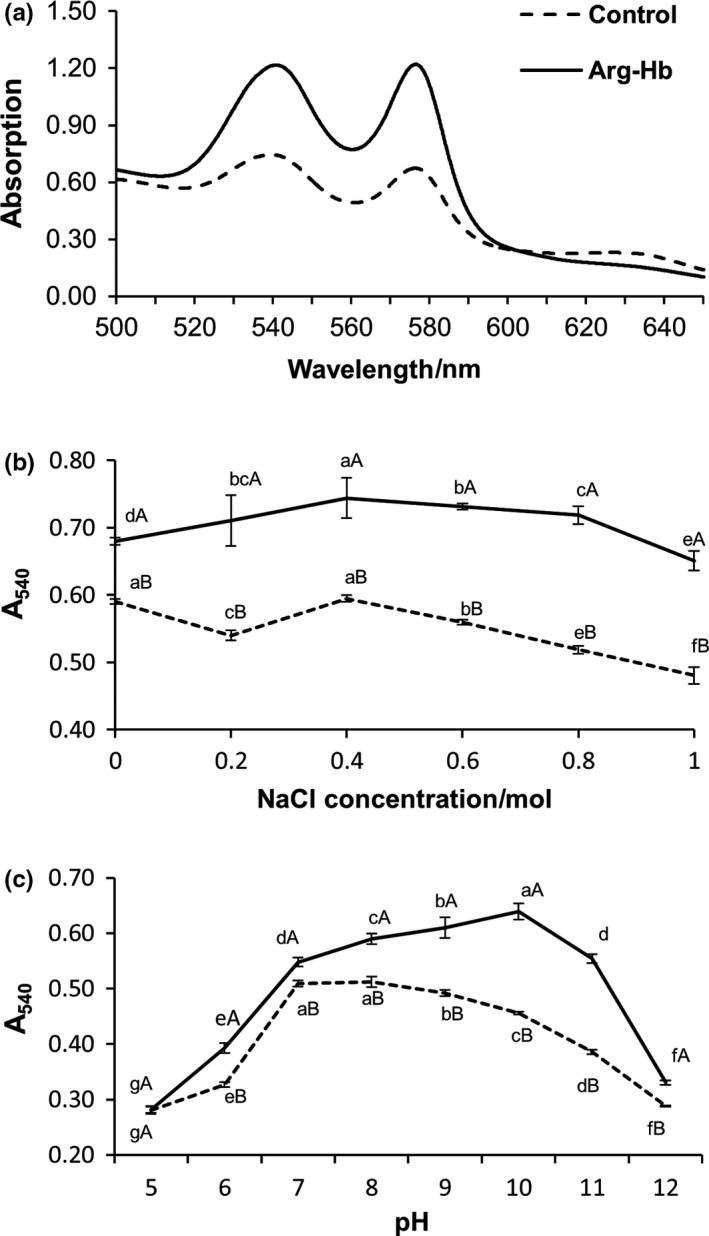
(a) Wavelength absorbance scan of prepared Arg‐Hb and Hb solutions. (b,c) Effects of pH and NaCl concentration on the stability of Hb. Different lowercase letters on the line chart represent significant differences in different pH or NaCl concentration treatments. Different capital letters on the line chart represent the significant differences at *p < *0.05 between the Arg‐Hb group and the control at the same pH or NaCl concentration. Mean ± *SD*,* n* = 3.

As shown in Figure 3a, the control and Arg‐Hb samples were scanned from wavelength 500 to 650 nm. Two absorbance peaks at 540 and 576 nm were detected for both samples, and another peak at 630 nm was detected for Arg‐Hb powder. This is consistent with previous studies (Salvador et al., 2009; Zhou et al., 2014). The addition of Arg did not change the maximum absorption wavelength, indicating the Hb did not coordinate with Arg. Therefore, the absorbance measured at 540, 576, and 630 nm were also used to determine the relative percentage of derivative forms of Hb in Arg‐Hb powder as in the literature (Benesch et al., 1973; Zhou et al., 2015).

The characteristic absorbance peak of Hb at 540 nm (A_540_) was used to evaluate the stability of Hb in the present study. As shown in Figure 3b, the A_540_ of Arg‐Hb samples was significantly higher than that of the control (*p* < 0.05), indicating that Arg‐Hb is more stable than Hb in NaCl solution. A_540_ of Arg‐Hb increased first and then decreased with the increase of NaCl concentration. For both Arg‐Hb and Hb, the A_540_ values decreased when NaCl increased from 0.4 to 1.0 mol/L.

It has been reported that Arg‐Hb has higher absorbance at 540 nm possibly because Arg enhanced the stability of Hb concentrates by coordinating with free iron (Zhou et al., 2015). Waugh (2004) reported that the metal ions in NaCl solution complexed with Hb to form stable ligands through ionic bonds under certain salt concentration. When the NaCl concentration is too high (>0.4 mol/L), the solubility of Hb reduces due to an increase in ionic strength, which results in a decrease in cross‐linking extent and an increase in repulsion among proteins. The stability of Hb reduced due to the structure change (Hegde, Henein, & Varma, 2003; Hurtado, Saguer, Toldrà, Parés, & Carretero, 2012).

The pH had a significant influence on the stability of Hb (*p* < 0.05) (Figure 3c). Both Arg‐Hb and Hb showed a similar changing pattern in absorbance at 540 nm when solution pH increased, which increased first and then decreased when pH increased from 5 to 12. The result showed that the percentage of oxygenated hemoglobin in Hb and Arg‐Hb solutions decreased under acidic or alkaline conditions. Wu et al. (2009) reported that hemoglobin depolymerized from tetramers into biopolymers in acidic or alkaline extracellular environment. The A_540_ of Arg‐Hb was higher than that of control from pH 6 to 12, indicating Arg‐Hb was more stable than Hb. Additionally, the control group had the best stability at pH 7, while the Arg‐Hb group showed the highest stability at pH 10. The reason might be related to arginine as a basic amino acid, which needs to be studied further.

### 3.4 Color stability of Arg‐Hb powder during storage

**Figure 4:**
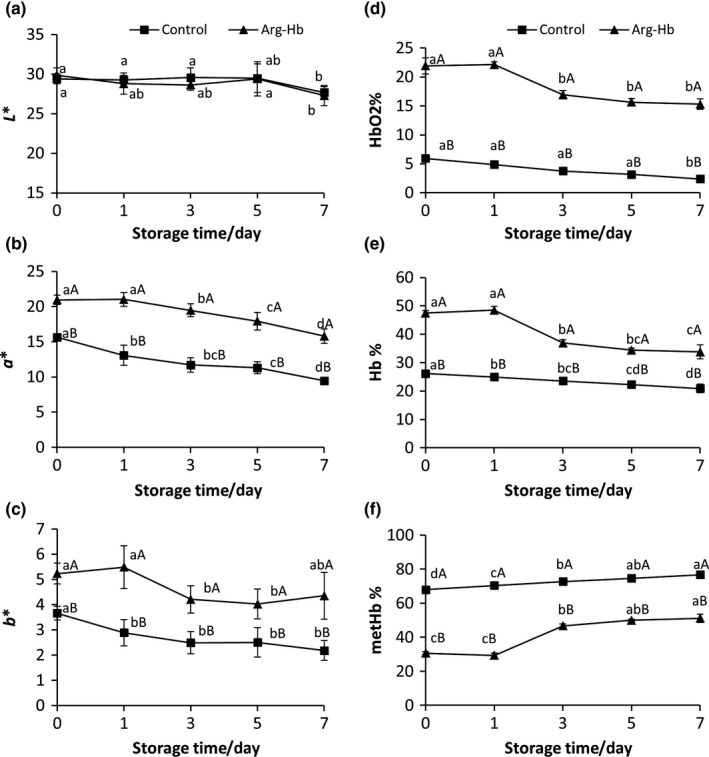
(a, b ,c) L*, a* and b* values of Arg‐Hb powder during storage. (d, e, f) HbO_2_%, Hb% and metHb% of Arg‐Hb powder during storage. Different capital letters on the line chart represent the significant differences at *p < *0.05 between the Arg‐Hb group and the control at the same storage time. Mean ± *SD*,* n* = 3

As shown in Figure 4a‐c, a* and b* values of Arg‐Hb samples were significantly higher than that of control during the whole storage period (*p* < 0.05), while no difference in L* values was observed between Arg‐Hb and control. The results showed that Arg‐Hb powder was more color stable than Hb powder during storage, indicating Arg enhanced the color stability of Hb. For both Arg‐Hb and Hb powder, a* and b* values decreased with the increase of storage time (*p* < 0.05). Similar results were also reported in previous studies when Hb was treated with l‐histidine, l‐cysteine, nicotinic acid, or nicotinamide (Saguer et al., 2003; Salvador et al., 2009; Zhou et al., 2014, 2015). These protective agents retard the oxidation of Hb, thus protecting the red color from browning. The safety of using protective agents is very important to the application of Hb powder. Numerous studies have shown that there are no safety concerns regarding Arg supplementation at an appropriate dose and chemical form (McKnight et al., 2010). Thus, Arg holds great promise as a safe and cost‐effective protective agent to stabilize Hb and making Hb more useful as a natural colorant.

Consistent with color measurement, the percentages of DeoxyHb and HbO_2_ were significantly higher (*p* < 0.05), but the oxidized gray‐brown MetHb was lower in Arg‐Hb powder than in control (Figure 4d‐f), further supporting that reaction with l‐Arg increased the color stability of Hb powder. For both Arg‐Hb and Hb powder, the percentages of DeoxyHb and HbO_2_ decreased and MetHb increased with the increase of storage time, showing Hb was oxidized during storage (*p* < 0.05), which was also observed in previous studies (Fontes et al., 2010; Zhou et al., 2015). In summary, the analysis of different forms of Hb showed that Arg inhibited the oxidation of Hb and increased color stability of Arg‐Hb powder during storage (Shahidi & Pegg, 1991). Nicotinic acid, nicotinamide, l‐cysteine, and l‐histidine have also been reported to prevent Hb from oxidation (Salvador et al., 2009; Zhou et al., 2014).

## 4 CONCLUSIONS

In this study, response surface methodology was used to determine the optimum technological conditions for Arg‐Hb preparation, which were determined to be Arg concentration of 10.5 mg/ml, reaction pH of 10.75, and temperature of 18°C. Under these conditions, the prepared Arg‐Hb powder contained 23.36% MetHb, which was close to the predicted value of 24.19%. The stability of Arg‐Hb powder was significantly higher than that of Hb powder in the presence of NaCl and at different pH conditions (*p* < 0.05). In addition, the Arg‐Hb powder had higher a* and b* values, and higher Hb and HbO_2_ content but lower MetHb content than control during storage. In summary, reaction with l‐Arg enhanced the color stability of Hb during freeze‐drying and storage.

## CONFLICT OF INTEREST

The authors declare that they do not have any conflict of interest.

## ETHICAL REVIEW

This study does not involve any human or animal testing.
